# Effect of Homebuyer Comment on Green Housing Purchase Intention—Mediation Role of Psychological Distance

**DOI:** 10.3389/fpsyg.2021.568451

**Published:** 2021-02-22

**Authors:** Qun Feng, Yan Wang, Chuanhao Chen, Zhengnan Dong, Xuejun Shi

**Affiliations:** ^1^Business School, University of Jinan, Jinan, China; ^2^School of Mathematics and Statistics, Shandong Normal University, Jinan, China

**Keywords:** green housing, purchase intention, psychological distance, homebuyer comment, green building

## Abstract

Green housing is a new type of building that advocates energy saving and environmental protection. How to stimulate buyers to buy green housing under the background of high cost is the key problem to guide green consumption. First of all, based on the existing literature, the comment of homebuyers was divided into comment quantity, comment quality, comment titer and evaluator credibility. The psychological distance mediation variable was introduced, and three dimensions of time distance, social distance, and space distance were selected to construct the influence model of homebuyer comment on green housing purchase intention. Meanwhile, the concept model was built, and questionnaires were adopted for empirical analysis. On this basis, considering the long-term purchase behavior of buyers, the influence model of homebuyers' second comment on green housing purchase intention with the Hotelling model was established. The results show that comment quality, comment titer, and the credit rating of the evaluator have a positive effect on green housing purchase intention while comment quantity has no significant effect. Psychological distance plays a mediation role between comment quality, comment titer, the credit rating of the evaluator, and green housing purchase intention while having a mediation effect between comment quantity and green housing purchase intention. In the long-term purchase behavior of green housing, psychological distance plays a greater role than price. At last, some suggestions were proposed.

## Introduction

Because of the increasingly serious environmental pollution in recent years, green housing has attracted much attention. People not only pursue the comfort of housing, but also prefer the housing which is green and healthy. Green housing has the advantages of energy saving and environmental protection, which is the type of building that the Chinese government strives to promote, but at the same time, it has the disadvantage of high prices, which makes the proportion of green housing in residential housing quite small. How to stimulate buyers to buy green housing and promote the growth of green building market is an important part of reducing energy consumption and protecting the environment.

When buying residential buildings, buyers mostly consider price, transportation cost and living convenience, etc., so the existing research explores the impacts of buyers' purchase behavior mainly from these factors. But with the improvement of living standards, a growing number of buyers, especially the green housing users who pursue a high quality of life, affected by environmental attitude, social influence, green house cognition, and so on, are more willing to share their living experience of green housing. Relevant research shows that homebuyer comment has an impact on green house purchase behavior, because the high price of green housing makes buyers more cautious and more willing to listen to the user experience to decide whether to buy. Some green-conscious buyers also prefer to make purchase decisions by referring to green housing users' share of whether the green houses are truly energy-efficient and environmentally friendly. However, there is a very limited amount of existing research exploring the impact of homebuyer comment on green housing purchase behavior. In fact, whether the comment of buyers can affect the purchase behavior of green housing depends on the degree of psychological recognition of the comment content of buyers. Evaluators with strong professional competence and good reputation have a greater positive impact on buyers, and the comment itself includes the content quality, the quantity, the titer, as well as the evaluator credibility will also affect the public praise and sales volume of the housing. The consumer's psychological cognition of the product will affect their processing degree of the information, and then lead to various consumption behaviors, for instance, Wang and Ding (2016) discussed the relationship between comment usefulness and psychological distance. Therefore, we introduce the theory of psychological distance to explore the impact of homebuyer comment on consumers' purchase intention to discuss how homebuyer comment influence the purchase behavior of green housing under the action of psychological distance, and considering the action of psychological distance, to explore the impact of the two-stage comment on green housing purchase behavior, thus to propose the suggestion for green housing consumption and green building development.

## Research Hypotheses

### Homebuyer Comment and Green Housing Purchase Intention

Under normal circumstances, the more the comment quantity, the stronger the consumer's purchase intention. When consumers make purchase decisions about green housing, according to Schiffman's consumption behavior pattern, the cognitive process is divided into three stages: demand recognition, pre-purchase search, and substitute comment. Wen (2012) believes that in the initial stage of housing consumption decision, consumers lack sufficient information to influence their purchase decision, and in order to collect as many product characteristics as possible about green housing, it is necessary to continuously search for the relevant information, which helps consumers break down their goals and thus increase their purchase intention and enjoy the true “value of green and low carbon.” Xue (2017) finds that there is a significant positive correlation between cognitive variables and behavioral will in green housing, that is, the more comprehensive the understanding of green housing, the higher the willingness to buy. Liu (2018) finds that the active or passive information of green housing has a significant positive effect on the purchase behavior of buyers, that is, the more sufficient the green housing information obtained is, the more it can improve the purchase intention of green housing buyers.

Hung (2017) points out that online comment quality includes comment sender objectivity, description integrity, and product richness. If the comment of its own quality is high, it can provide valuable advice for consumers, thus positively affecting consumers' purchase intention. Yang et al. (2019) propose that the internalized green housing characteristic information, specific income and quality assurance information can improve the green housing purchase intention.

Comment titer refers to the direction of comment content, including three dimensions: positive, neutral, and negative. A positive comment will let consumers perceive positive emotional tendency to products or services. A negative comment lets consumers perceive negative emotional tendency, while for a neutral comment, consumers do not perceive any emotional tendency. Behavioral attitude refers to the positive or negative comment of a commodity by consumers. Xue (2017) decomposes the behavior attitude into: economy, residence attitude, and environmental protection attitude to study the influence of these three factors on the consumption behavior of green housing, and finds that there is a significant positive correlation between these three factors and behavior intention. Yang and Wu (2017) point out that cognitive psychology has a positive effect on green housing purchase decisions by studying the influencing factors of green housing choice behavior, while the theory of planning behavior holds that the individual's positive or negative comment of a certain behavior is based on the consumer's cognitive degree. When the comment titer is positive, the more significant the external stimulus, the easier it is for consumers to form a positive purchase attitude. The purchase attitude has a positive influence on the green housing purchase intention, that is, the buyers consider that the green housing purchase behavior is correct and wise, so they are willing to buy (Li, 2016).

As to the evaluator's credibility, some scholars divide it into the evaluator's professionalism and reliability. Professionalism refers to the extent of consumer's perception to the correctness of evaluator's information. Schiffman and Kanuk (1997) believe that when the comment publisher is highly professional, it will reduce the perception risk of consumers to the product and increase the sense of trust, thus affecting the consumer's purchase intention. The reliability of the evaluator refers to the consumer's perception of the evaluator's reliability, which has nothing to do with the comment content itself (Huang Q. P., 2016). In 2006, China launched the green building comment standard, the implementation of this standard has promoted the development of the green housing construction industry in China. For green housing with government certification, consumers' purchase intention is obviously higher than that of ordinary green housing (Xue, 2017). Yang et al. (2019) believe that government agencies and third-party certification agencies have a positive impact on consumers' reception of green housing quality assurance information, which affects their value perception and changes their consumption decisions. On January 1, 2015, the Chinese government implemented the new edition of the Green Building comment Standard, which regulated the market of green housing, improved the performance of green housing, and then increased its sales. Li (2016) thinks that because of the asymmetry of information, buyers have certain pressures when choosing green housing, while the government's green housing certification logo has a certain authority and systematization, which maximizes the benefit of reference information, thus affecting the buyers' purchase intention. In summary, the following hypotheses were proposed:

H1: Homebuyer comment is divided into four dimensions: comment quantity, comment quality, comment titer, and evaluator credibility.

H1a: Comment quantity has a positive effect on green housing purchase intention.

H1b: Comment quality has a positive effect on green housing purchase intention.

H1c: When the comment titer is positive, the purchase intention of green housing increases.

H1d: Evaluator credibility has positive effect on green housing purchase intention.

### Relationship Between Homebuyer Comment and Psychological Distance

#### Comment Quantity and Psychological Distance

Fiedler (2007) thinks that the degree of professionalism of the individual to receive information can improve the efficiency of the individual to process information. The more product information the individual understands, the higher the degree of professionalism, the less time and effort is needed to process the information. According to the interpretation level theory (Bar-Anan et al., 2007), the more information the individual understands and the more detailed it is, the theory of low interpretation level is applicable to interpretation, which means that the psychological distance will also be closer. Li (2016) concludes in the study of the influence mechanism of green housing purchase intention: the more consumers understand the product knowledge, the stronger his/her ability to identify the product, the higher the degree of cognition, the closer the psychological distance, and the more conducive to the formation of product value and to the preference emotion judgment, which will further improve the green housing purchase intention. The quantity of buyers mastering green characteristic information has an important influence on their purchase intention of green products, and the wider they master the information, the deeper the cognitive degree, the closer the psychological distance, the stronger the willingness to buy (Yang et al., 2019).

The concept of “psychological distance” studied in this paper was proposed by Liberman and Trope in his study of social psychology in 1988. It refers to the subjective experience of an event from the moment, this place, itself, and the event's possible occurrence. Trope and Liberman in 2007 proposed four dimensions of psychological distance that affect consumer decision-making, namely: time distance, social distance, spatial distance, and hypothesis (Bar-Anan et al., 2007). People have different attitudes toward things that will happen at different time distances. Individuals usually think of things that will happen in the far future as abstract, far away from themselves, and tend to show a more positive and optimistic attitude. What is going to happen in the near future is specific, close to oneself, it takes time to think, usually showing a negative and pessimistic attitude (Trope and Liberman, 2000). Social distance refers to that based on the self, the individual's perception of the distance between the subject and the self. Trope and Liberman (2008) studied the relationship between social distance and the level of interpretation, and pointed out that because psychological distance is a self-centered concept, the more similar and close to the individual, the closer the psychological distance between others and the individual; Conversely, the more obvious the difference between individuals, the lower the degree of intimacy, the greater the psychological distance between individuals. Spatial distance refers to the individual's perception of the distance between events or objects in space. Fujita et al. (2006) found that when people think about things that are far away from them in space, they are more willing to use abstract language to describe these events, corresponding to a high level of interpretation. Probability is independent of time, social and spatial distance. Its level can cause people's psychological distance to change. In a small probability situation, people's high level of interpretation is activated, which tends to lead to generalized or abstract descriptions of events. In high-probability situations, people's low level of interpretation is activated, and they tend to describe events in detail (Wakslak et al., 2006).

Four subdimensions of psychological distance: time distance, social distance, spatial distance, and hypothesis have been proved highly correlated (Zhu, 2018). So based on the above analysis, it is inferred that comment quantity has a negative effect on the psychological distance of consumers as well as on the four subdimensions of psychological distance. Based on the above research, the following hypotheses are proposed:

H2a: Comment quantity has a negative effect on time distance

H2b: Comment quantity has a negative effect on social distance

H2c: Comment quantity has a negative effect on spatial distance.

#### Comment Quality and Psychological Distance

Because online shopping is anonymous and virtual, that is, the physical goods cannot be touched, consumers can only perceive the quality of goods through comments, and the quality of these comments directly affects their trust in products. Especially, the more negative comments, the greater the impact (Huang L., 2016). A high-quality comment, is one which contains more explicit views, and more detailed examples and information. According to cognitive psychology, the more specific the information of things, the easier it is to improve people's perceptual sensitivity, and the closer the psychological distance perception is (Zhou et al., 2015). Zeng (2008) pointed out in his study that the quality of comment affects whether subsequent buyers get useful information. Park et al. (2007) divided the comment quality into four indicators: relevance, comprehensibility, adequacy, objectivity. They point out that high-quality comments, with strong logic and clear persuasion, will shorten the psychological distance with consumers through sufficient and objective reasons. On the contrary, a low-quality comment has subjective and emotional color, and lacks the basis to convince consumers. Godes and Mayzlin (2004) find that comment quality also has a positive impact on product sales. Jiang et al. (2015) thinks that buyers' preference for green value and basic value of green housing have an impact on their purchase behavior, which affects consumption choice by influencing personal perception. When the green value in the comment is more than the basic value, the comment has higher quality. Wang and Liu (2018) point out that there is information asymmetry in the green housing market, and whether buyers can obtain reliable relevant information will directly affect their cognition of green housing products, and then affect the purchase decision. In summary, the following hypotheses were proposed:

H3a: Comment quality has a negative effect on time distance.

H3b: Comment quality has a negative effect on social distance.

H3c: Comment quality has a negative effect on spatial distance.

#### Comment Titer and Psychological Distance

Pumawirawan et al. (2012) find that the titer of a comment can be expressed as three dimensions: positive, neutral, or negative. Since a neutral comment does not have any emotional color and descriptive information on both positive and negative sides, the comment titer is directly divided into positive and negative. The theory of persuasion effect points out that a comment can cause the change of consumers' attitude toward the product, and then affect the change of psychological distance to the product, that is, the comment titer will affect the quantity of sales (Xiao, 2019). Positive comments will shorten the psychological distance between consumers and products, promoting the desire to buy, while negative comment will weaken consumers' trust in products, increase psychological distance, and thus reduce consumption motivation. Song (2008) argues that if an individual is positive about green life, then they are more likely to participate in green product purchase and consumption activities. Therefore, enterprises should spread the comment information about the positive health of green housing in propaganda to promote consumption behavior. Based on the above research, the following hypotheses were proposed:

H4a: When the titer is positive, the time distance is shortened.

H4b: When the titer is positive, the social distance is shortened.

H4c: When the titer is positive, the spatial distance is shortened.

#### Credibility of the Evaluator and Psychological Distance

Zhang (2016) pointed out in the study that the certification of green buildings by third-party authorities has a positive effect on the willingness to buy. The Chinese Regulation on Accreditation stipulates that the third-party certification body of green building is approved by the supervision and administration department of the State Council and has strong credibility and persuasion. Some scholars believe that the comment of green buildings by the government plays an important role in the purchase of consumers. Because the comment system of the government is specific, standardized, and systematic, and the comment content is authoritative, this will shorten the psychological distance of consumers and then affect the purchase decision. An important factor affecting the credibility of information is the source of information, thus as a source of information, the evaluator's own credibility is crucial. Zhou (2009) divides the credit rating of the evaluator into the professional ability and reliability, and points out that the credit rating of the evaluator affects the brand decision by influencing the brand comment. There is the same impact in other areas, as found in research in the film industry, where expert comment is positively correlated with film box office performance. Liu (2018) believes that there are obvious differences between green housing and traditional “brown housing,” and only when buyers understand and accept this difference, can they reduce the psychological distance. The official certification of the government can strengthen the authenticity of the information and promote the interest and even the trust of buyers, thus affecting the purchase intention. Based on the above studies, the following hypotheses were proposed:

H5a: Credibility of the evaluator has a negative effect on time distance.

H5b: Credibility of the evaluator has a negative effect on social distance.

H5c: Credibility of the evaluator has a negative effect on spatial distance.

### Relationship Between Psychological Distance and Green Housing Purchase Intention

Liberman and Trope first linked the concept of psychological distance to the theory of interpretation level in 1998. They proposed four dimensions of psychological distance in 2007: time distance, social distance, spatial distance, and probability of event occurrence.

Because online comment is mostly anonymous, with strong uncertainty, buyers cannot experience it personally when visiting the comment, so time distance and social distance are mostly used for the exploration of online comment in online marketing (Kang, 2017). On this basis, we add space distance to study the relationship between homebuyer comment and green housing purchase intention.

Li (2019) studies the influencing factors of green product purchase intention based on consumer value theory, and concludes that under the regulation of time distance (psychological distance), the high interpretation level of consumers is aroused, which makes them more inclined to pay attention to essential and core characteristics, and thus more willing to buy green products. Wang et al. (2017) point out that green appeal advertising in green purchase decision-making process can be divided into rational appeal and emotional appeal by studying the influence mechanism of green emotion appeal on green purchase decision-making process, in which emotional appeal has a more significant impact. Wang (2017) pointed out that the psychological distance between consumers and green products is different in different problem situations when studying the psychological mechanism of green consumption attitude-behavior separation. This kind of psychological distance is mainly time dimension. In terms of time dimension, in attitude comment stage, the psychological distance between consumers and green products is far away.

#### Time Distance and Green Housing Purchase Intention

Cheng (2017) thinks that the high entry degree commodity has high capital value, and many factors need to be considered. Corresponding to the theory of high interpretation level, the farther the time distance, the higher the purchase decision, whereas the low entry degree commodity only emphasizes the feasibility demand. On the contrary, corresponding to the low interpretation level theory, the closer the time distance, the higher the purchase decision. In the case of close-time distance, price information is a representation of expenditure perception to consumers, and high price means higher money expenditure, so the purchase intention will decrease; while in the case of far-time distance, it is a representation of quality perception, and the higher price represents the better quality, and the higher purchase intention (Zhang and Zhao, 2016). Wen (2012) points out that with high house prices in the past 2 years, consumers are not willing to pay higher costs for green housing. According to traditional economic theory, green housing will bring long-term benefits to consumers.

H6a: Time distance has a positive effect on green housing purchase intention.

#### Social Distance, Spatial Distance, and Green Housing Purchase Intention

The explanation of behavior theory points out that the change of psychological distance will affect the change of consumer decision. The physical position of the buyer and seller will affect the buyer's purchase decision. The distance of space leads to the psychological alienation between people, which leads to long social distance and reduces the buyer's trust in the seller (Huang and Zhao, 2013). Wang et al. (2017) point out that the pride dimension has the most significant influence on green purchase behavior, that is, the closer the consumer society distance is, the more willing they are to take more responsibility for the society, and the higher the willingness to buy green products. “Space discount” is derived from the concept of spatial distance, which shows that the farther the spatial distance is, the lower the actual utility is, and the lower the actual utility is perceived by people (Ma, 2016). When buyers buy green housing, the relevant policies of the government and the support degree of family and friends have a positive relationship with the purchase intention, indicating that the closer the social distance, the higher the purchase intention (Xue, 2017).

H6b: Social distance has a negative effect on green housing purchase intention.

H6c: Spatial distance has a negative effect on green housing purchase intention.

### Effect of Psychological Distance on the Relationship Between Homebuyers Comment and Green Housing Purchase Intention

In the context of online shopping, consumer comment is also called “online comment,” “online word of mouth” and so on (Deng, 2018). In terms of influencing factors of consumer shopping, according to the theory of interpretation behavior, individual decision-making is vulnerable to the influence of matching information with the level of interpretation it is in, so psychological distance can play a role in decision-making results through the level of interpretation (Li and Liu, 2020). Based on the above literature analysis, it is found that when the comment titer is positive, the higher the comment quantity, the comment quality, and evaluator credibility, and the closer the consumer psychological distance. The closer the consumer psychological distance (social distance, spatial distance), the higher the green housing purchase intention. The following hypotheses were therefore proposed:

H7a: Psychological distance plays a mediation role between the comment quantity and the green housing purchase intention.

H7b: Psychological distance plays a mediation role between the comment quality and the green housing purchase intention.

H7c: Psychological distance plays a mediation role between evaluator credibility and the green housing purchase intention.

H7d: When the comment titer is positive, psychological distance plays a mediation role between comment titer and the green housing purchase intention.

**Figure 1:**
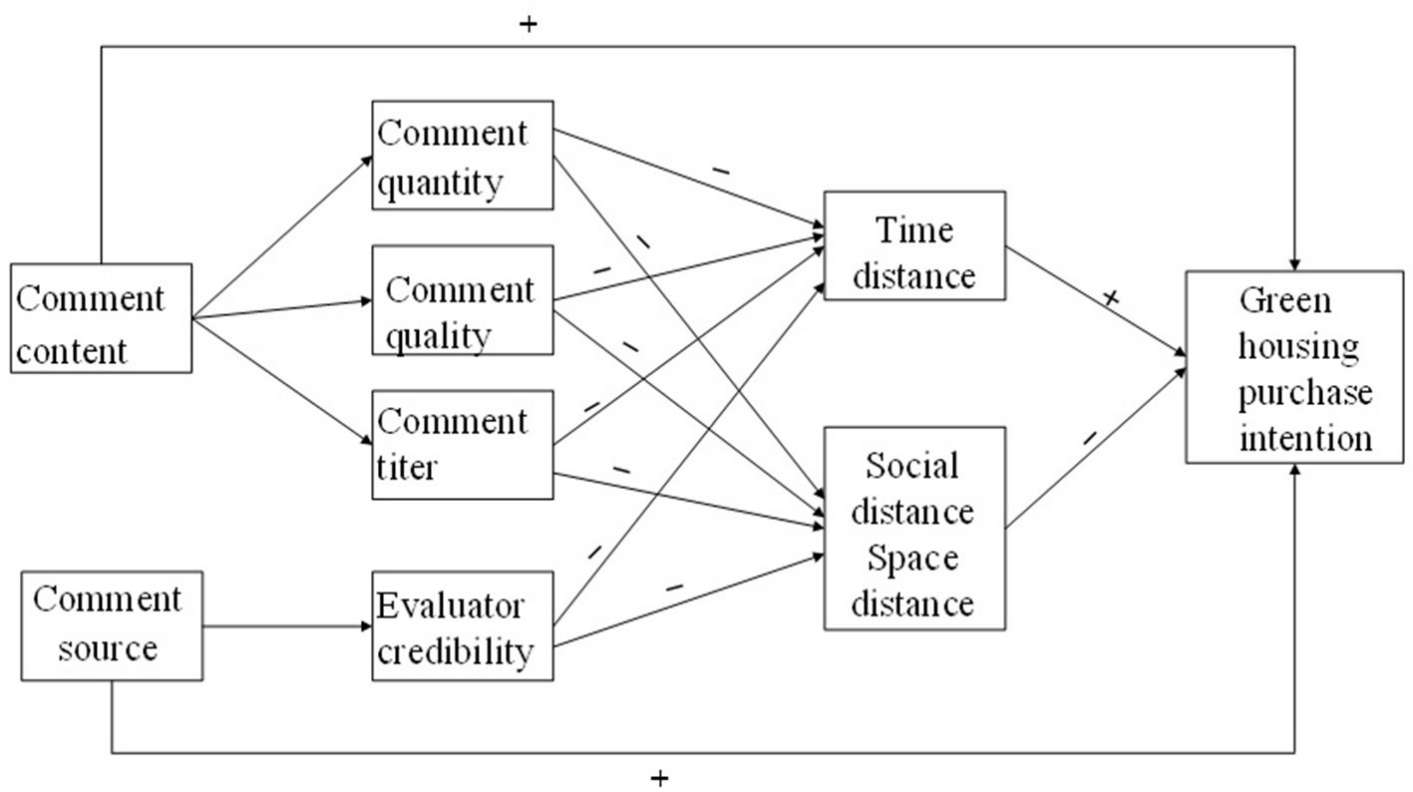
Conceptual model.

To sum up, the conceptual model is shown in Figure 1.

## Questionnaires and Research

### Definition and Measurement of Variables

#### Definition of Variables

On the basis of summarizing the existing literature and the research requirement, the variable definition of the research model was carried out. As an independent variable, its four dimensions are comment quantity, comment quality, comment titer, and evaluator credibility. As a mediation variable, the three dimensions of psychological distance are time distance, social distance, and spatial distance. The dependent variable is green housing purchase intention. The definitions and references of the variables are shown in Table 1.

**Table 1:** Definition of variables.

	**Variables**	**Definition**	**References**
Independent variables	Comment quantity	Comment quantity can enrich the comment information and increase the total amount of information.	Liu and Tang (2016)
	Comment quality	The description integrity and product richness of the comment information, the objectivity of the comment sender, whether or not valuable information was provided	Hung (2017) and Park et al. (2007)
	Comment titer	Direction of the comment content, including positive, neutral, negative	Pumawirawan et al. (2012)
	Evaluator credibility	Evaluator's professionalism and reliability	Schiffman and Kanuk (1997)
mediation variables	Time distance	Time distance refers to people's perception of events about time.	Bar-Anan et al. (2007)
	Social distance	Based on the self, the individual's perception of the distance between the subject and the self.	Fujita et al. (2006)
	Spatial distance	It refers to an individual's spatial or geographical proximity between himself/herself and the reference object.	Ma (2016)
Dependent variable	Green housing purchase intention	Effect of the comment on the information recipient's willingness to buy	Bansal and Voyer (2000)

#### Measurement of Variables

On the basis of the mature scale, slight adjustment and modification were made to the scales according to the actual situation. The questionnaire was measured using the Likert 5 score scale to set options. “1” represents considerable disapproval, “2” represents disapproval, “3” represents neutrality, “4” represents endorsement, and “5” represents considerable endorsement.

(1) The homebuyer comment scale

According to the design of Zhang (2016), the homebuyer comment scale was designed using four dimensions: comment quantity, comment quality, comment titer, and evaluator credibility. The scale is shown in Table 2.

**Table 2:** The homebuyer comment scale.

**Variables**	**Dimensions**	**Questions**	**Source**
Homebuyer comment	Comment quantity	With regard to green housing, buyers have published a lot of reviews	Park and Kim (2007) and Bi (2010)
		With regard to green housing, these comments provide a great deal of information	
		A lot of people evaluate on the buying platform	
	Comment quality	The comments of green housing are basically objective	Jin (2007)
		Most comments are easy to understand	
		These comments give me a better understanding of the performance of green housing	
	Comment titer	The comments of green housing are generally positive	Park et al. (2007)
		The overall recommendation of the comment is to purchase the green housing	
		The comments are mostly positive for green housing.	
	Evaluator credibility	Some evaluators have expertise related to green housing	Bansal and Voyer (2000)
		Most evaluators have a higher level of online shopping	
		Most evaluators are credible

(2) Psychological distance scale

For the measurement of psychological distance, the scales of Xv and Li (2018), Huang and Zhao (2013) were referred to, and combined with the needs of practical research, the scale is designed as Table 3.

**Table 3:** Psychological distance scale.

**Variables**	**Dimensions**	**Questions**	**Source**
Psychological distance	Time distance	These comments give me specific information about green housing in a short-term	Huang and Zhao (2013)
		These comments make me want to buy green housing in the future.	
	Social distance	These comments made me feel like I was in a similar context to the evaluator	Huang and Zhao (2013)
		These comments give me a better idea of a brand of green housing	
		After reading these comments, I would like to buy green housing for others or help others to buy green housing	
	Spatial distance	These comments make me feel that the journey to a green housing is acceptable	Huang and Zhao (2013) and Xv and Li (2018)
		After reading these comments, I want to buy green housing in this city

(3) Purchase intention scale

By referring to the scale of Bansal and Voyer (2000), the purchase intention scale is designed as Table 4.

**Table 4:** Purchase intention scale.

**Variables**	**Questions**	**Source**
Purchase intention scale	After reading these comments, I would like to try to use green housing	Bansal and Voyer (2000)
	These comments affect whether I choose to buy the product	
	I would recommend green housing to others	
	I'm very likely to buy a green house in the future

(4) Demographic variables

Demographic variables refer to the basic elements of the questionnaire survey to distinguish the group of homebuyers, mainly gender, age, education level, professional background, and monthly income level, etc.

### Structure and Empirical Analysis of the Questionnaire

The questionnaire consists of two parts, the first part is the research control variables, including the subject's gender, age, education, occupation, monthly income, etc. The second part is the survey homebuyer comment scale, psychological distance scale, and green housing purchase intention scale. There are 28 questions in the questionnaire, including 5 questions for control variables, 12 questions in homebuyer comment scale, 7 questions in psychological distance scale, and 4 questions in purchase intention scale.

The questionnaires were distributed through the website of Questionnaire Star to obtain data. A total of 368 questionnaires were collected, in which 102 invalid and repeated questionnaires were excluded, 266 valid questionnaires were adopted, and the effective rate of the questionnaires was 72.28%. The study was approved by the Ethics Review Board of Business School in University of Jinan, China. All participants provided informed consent in compliance with the Declaration of Helsinki before the survey. The SPSS23.0 software was used for analysis.

The descriptive statistics about the file survey of this paper are shown in Table 5.

**Table 5:** Descriptive analysis of basic information.

**Basic information**	**Classification**	**Frequency**	**Percentage**
Gender	Male	121	45.5
	Female	145	54.5
Age	Under 18	4	1.5
	Between 19 and 20	143	53.8
	Between 30 and 39	68	25.6
	Over 40	51	19.2
Education	Junior high school/high school/technical secondary school	48	18.0
	Junior college	41	15.4
	Undergraduate	153	57.5
	Master	24	9.0
Profession	College students	76	28.6
	Staff	84	31.6
	Freelance	73	27.4
	Government or public institution employees	33	12.4
Monthly income	3,000~5,000	114	42.9
	5,000~8,000	55	20.7
	8,000~15,000	69	25.9
	Over 15,000	28	10.5

#### Reliability Analysis

Reliability is used to test the stability and consistency of the questionnaire results in the measurement of the relevant variables. Reliability can reflect whether the questionnaire has credibility. The internal consistency index is used to test the reliability of the scale. The values of each scale are shown in Table 6.

**Table 6:** Reliability test results.

**Scales**	**Research variables**	**Cronbach α coefficients**
Homebuyer comment	Comment quantity	0.804
	Comment quality	0.742
	Comment titer	0.763
	Evaluator credibility	0.738
Psychological distance	Time distance	0.630
	Social distance	0.806
	Spatial distance	0.718
Green housing purchase intention	Green housing purchase intention	0.838

It can be seen that the reliability of the evaluation quantity is 0.804, the reliability of the evaluation quality is 0.742, the reliability of the evaluation titer is 0.763, the reliability of evaluator credibility is 0.738, the reliability of the time distance is 0.630, the reliability of the social distance is 0.806, the reliability of the space distance is 0.718, and the reliability of the green housing purchase intention is 0.838. The results indicate that the data reliability of this questionnaire is high and can be further analyzed.

#### Validity Analysis

Validity is the accuracy and effectiveness of the questionnaire, that is, the correctness of measuring a certain behavior characteristic. When the scale can accurately measure the required measurement content, it shows that this measurement is effective. The statistical indexes of validity were KMO (validity) and the significance of Bartlett Spherical Test. The results are shown in Table 7.

**Table 7:** KMO and Bartlett results of the questionnaires.

Homebuyer comment scale	KMO	0.918	
	Bartlett spherical test	Approximate chi-square	1389.010
		Degree of freedom	66
		Sig.	0.000
Psychological distance scale	KMO	0.901	
	Bartlett spherical test	Approximate chi-square	848.059
		Degree of freedom	21
		Sig.	0.000
Green housing purchase intention scale	KMO	0.818	
	Bartlett spherical test	Approximate chi-square	395.119
		Degree of freedom	6
		Sig.	0.000

Table 7 shows that the KMO value of homebuyer comment is 0.918, the KMO value of psychological distance is 0.901, and the KMO value of green housing purchase intention is 0.818. All the results are larger than 0.8, and the Sig. values of the Bartlett spherical test are all smaller than 0.05, so the structural validity of the three scales is good, and they can be further analyzed.

#### Correlation Analysis

Correlation analysis is to analyze the correlation between the research objects, and to explore the degree of closeness of their relationship. Pearson correlation coefficient was adopted to judge the correlation between each two of comment quantity, comment quality, comment titer, evaluator credibility, time distance, social distance, spatial distance, homebuyer comment, psychological distance, and green housing purchase intention. The value of the correlation coefficient “r” is [−1, and the greater the absolute value of “r,” the more significant the correlation is. The correlation analysis results are shown in Table 8.

**Table 8:** Correlation results analysis.

	**Comment quantity**	**Comment quality**	**Comment titer**	**Evaluator credibility**	**Time distance**	**Social distance**	**Spatial distance**	**Homebuyer comment**	**Psychological distance**	**Green housing purchase intention**	
Comment quantity	Pearson correlation	1									
Comment quality	Pearson correlation	0.625^**^	1								
Comment titer	Pearson correlation	0.562^**^	0.604^**^	1							
Evaluator credibility	Pearson correlation	0.562^**^	0.609^**^	0.732^**^	1						
Time distance	Pearson correlation	−0.512^**^	−0.621^**^	−0.606^**^	−0.726^**^	1					
Social distance	Pearson correlation	−0.567^**^	−0.598^**^	−0.689^**^	−0.750^**^	0.707^**^	1				
Spatial distance	Pearson correlation	−0.503^**^	−0.488^**^	−0.623^**^	−0.650^**^	0.599^**^	0.743^**^	1			
Homebuyer comment	Pearson correlation	0.824^**^	0.843^**^	0.850^**^	0.857^**^	−0.729^**^	−0.769^**^	−0.669^**^	1		
Psychological distance	Pearson correlation	−0.594^**^	−0.640^**^	−0.720^**^	−0.797^**^	0.864^**^	0.919^**^	0.882^**^	−0.813^**^	1	
Green housing purchase intention	Pearson correlation	0.499^**^	0.528^**^	0.594^**^	0.648^**^	−0.666^**^	−0.698^**^	−0.774^**^	0.671^**^	−0.804^**^	1

* P <0.05;

** P <0.01;

*** *P <0.001*.

According to the results of the Pearson correlation analysis in Table 8, the pairwise correlations of comment quantity, comment quality, comment titer, evaluator credibility, time distance, social distance, spatial distance, homebuyer comment, psychological distance, and green housing purchase intention are all at the 0.001 significance level, which can be further inspected by regression test.

#### Regression Analysis

The impact of comment quantity, comment quality, comment titer, and evaluator credibility on time distance, social distance, spatial distance, homebuyer comment, psychological distance, and green housing purchase intention, as well as its degree were inspected by the regression analysis. The results are shown in Table 9.

**Table 9:** Regression analysis on homebuyer comment to psychological distance and on psychological distance to purchase intention.

**Dependent variable**	**Time distance**	**Social distance**	**Spatial distance**	**Green housing purchase intention**
	**Model 1**	**Model 2**	**Model 3**	**Model 4**
Gender	−0.013	0.023	−0.005	−0.068
Age	−0.040	−0.071	0.008	−0.135^***^
Profession	−0.026	0.026	−0.011	−0.028
**Independent variables**				
Comment quantity	−0.035	−0.127^*^	−0.133^*^	
Comment quality	−0.251^***^	−0.115^*^	−0.019	
Comment titer	−0.065	−0.216^***^	−0.271^***^	
Evaluator credibility	−0.507^***^	−0.449^***^	−0.366^***^	
Time distance				−0.271^***^
Social distance				−0.134^*^
Spatial distance				−0.511^***^
*R*^2^	0.583	0.634	0.482	0.689
*F*	51.501^***^	63.871^***^	34.313^***^	95.505^***^
Δ*R*^2^	0.572	0.624	0.468	0.682
Df	7	7	7	6

n = 266, standardized regression coefficients are presented in the table:

* P <0.05;

** P <0.01 being significant in terms of level;

*** *P <0.001*.

It can be seen that in Model 1, the regression analysis of comment quantity, comment quality, comment titer, and evaluator credibility to time distance shows that the *F* value is 51.501, the significance is <0.001, the regression model is significant, and the adjusted *R*^2^ is 0.572. The influence of comment quality and evaluator credibility on time distance reach a significant level, and the regression coefficients were −0.251 and −0.507, respectively. The effect of comment quantity and comment titer on time distance is not significant. Therefore, the hypotheses H3a and H5a are valid, while H2a and H4a are not.

In Model 2, the regression analysis of comment quantity, comment quality, comment titer, and evaluator credibility to social distance shows that the F value is 63.871, the significance is less than 0.001, the regression model is significant, and the adjusted *R*^2^ is 0.624. The influence of comment quantity, comment quality, comment titer, and evaluator credibility on social distance all reach a significant level, and the regression coefficients were −0.127, −0.115, −0.216, and −0.449, respectively. Therefore, the hypotheses H2b, H3b, H4b, and H5b are valid.

In Model 3, the regression analysis of comment quantity, comment quality, comment titer, and evaluator credibility to spatial distance shows that the *F* value is 34.313, the significance is <0.001, the regression model is significant, and the adjusted *R*^2^ is 0.468. The influence of comment quantity, comment quality, comment titer, and evaluator credibility on spatial distance reach a significant level, and the regression coefficients were −0.133, −0.271, and −0.366, respectively. The effect of comment quality on spatial distance is not significant. Therefore, the hypotheses H2c, H4c, H5c are valid, while H3c is not.

In Model 4, the regression analysis of time distance, social distance, and spatial distance to green housing purchase intention shows that the *F* value is 95.505, the significance is <0.001, the regression model is significant, and the adjusted *R*^2^ is 0.682. The influence of time distance, social distance, and spatial distance on green housing purchase intention all reach a significant level, and the regression coefficients were −0.271, −0.134, and −0.511, respectively. Therefore, the hypotheses H6b and H6c are valid, but the regression analysis of time distance to green housing purchase intention shows that time distance has a negative effect on green housing purchase intention. Therefore, H6a is not valid.

#### Mediation Effects

The mediation effect of psychological distance is tested in this section. The premise of mediation effect is to analyze the mediation variable when the relationship between independent variable and dependent variable is significant, otherwise there will be no mediation effect. The first step: conduct regression analysis with taking comment quantity, comment quality, comment titer and evaluator credibility as independent variables, and the green housing purchase intention as a dependent variable. The second step: conduct regression analysis with taking comment quantity, comment quality, comment titer, and evaluator credibility as independent variables, and psychological distance as dependent variable. The third step: conduct regression analysis with taking comment quantity, comment quality, comment titer, evaluator credibility, and psychological distance as independent variables, and the green housing purchase intention as dependent variable, and then carry out a mediation effect test. The results are shown in Table 10.

**Table 10:** Mediation effect test of psychological distance on homebuyer comment and purchase intention.

**Dependent variables**	**Green housing purchase intention**	**Psychological distance**	**Green housing purchase intention**
	**Model 1**	**Model 2**	**Model 3**
Gender	−0.069	0.002	−0.068
Age	−0.118^*^	−0.038	−0.147^***^
Profession	−0.016	−0.004	−0.019
**Independent variables**			
Comment quantity	0.101	−0.111^*^	0.014
Comment quality	0.130^*^	−0.143^**^	0.018
Comment titer	0.190^**^	−0.208^***^	0.028
Evaluator credibility	0.370^***^	−0.495^***^	−0.016
Mediation variable			
Psychological distance			−0.781^***^
*R*^2^	0.490	0.703	0.671
F	35.381^***^	87.348^***^	65.490^***^
Δ*R*^2^	0.476	0.695	0.661
Df	7	7	8

n = 266, standardized regression coefficients are presented in the table:

* P <0.05;

** P <0.01 being significant in terms of level;

*** *P <0.001*.

According to Table 10, in step 1, the regression analysis of comment quantity, comment quality, comment titer, and evaluator credibility to green housing purchase intention shows that the *F* value is 35.381, the significance is <0.001, the regression model is significant, and the adjusted *R*^2^ is 0.476. The influence of comment quality, comment titer, and evaluator credibility on green housing purchase intention reach a significant level, and the regression coefficients were 0.130, 0.190, and 0.370 respectively. The effect of comment quantity on green housing purchase intention is not significant. Therefore, the hypotheses H1b, H1c, and H1d are valid, while H1a is not.

In step 2, the regression analysis of comment quantity, comment quality, comment titer, and evaluator credibility to psychological distance shows that the *F* value is 87.348, the significance is <0.001, the regression model is significant, and the adjusted *R*^2^ is 0.695. The influence of comment quantity, comment quality, comment titer, and evaluator credibility on psychological distance all reach a significant level, and the regression coefficients were −0.111, −0.143, −0.208, and −0.495.

In step 3, the regression analysis of comment quantity, comment quality, comment titer, evaluator credibility, and psychological distance to green housing purchase intention shows that the *F* value is 65.490, the significance is <0.001, the regression model is significant, and the adjusted *R*^2^ is 0.661. The influence of psychological distance on green housing purchase intention reaches a significant level, and the regression coefficients were −0.781. With the addition of mediation variables, the influence of comment quantity, comment quality, comment titer, and evaluator credibility on green housing purchase intention all fail to reach a significant level. So, the mediation effect of psychological distance on comment quantity, comment quality, comment titer, evaluator credibility, and green housing purchase intention is complete mediation. Therefore, the hypotheses H7b, H7c, and H7d are valid, while H7a is not.

## Green Housing Selection Model Based on Homebuyer Comment

In order to further study green housing buying behavior, this section considers the determinants of multi-stage buying behavior based on homebuyer comment from a long-term perspective.

### Basic Model

(1) Homebuyers with the same income will get the same utility when they buy the satisfactory buildings.
(2) Psychological distance will increase their utility;
(3) The cost of homebuyers includes transportation costs and housing costs.

When consumers want to buy buildings, they will consider the distance to their workplace or common service areas (collectively referred to as the service area), such as hospitals and supermarkets, and the transportation costs are taken into the cost of housing. Using the Hotelling model, one green housing and one ordinary building's zi are included in the model with a distance of 1. The distance between the service area and the green housing is x, and the distance between the service area and the ordinary buildings is 1–x.

The homebuyer's utility function can be obtained:

$$\begin{array}{l} U_{i}=V\;+\;kw_{i}\;-\;t|{x\;-\;z}_{i}|-p_{i} \end{array}$$

V represents the homebuyers' utility when satisfying their basic housing needs; ***w***_***i***_ represents the homebuyer's psychological distance; ***kw***_**i**_ represents the utility of homebuyer's psychological distance; ***t***|***x*−*z***_***i***_| denotes the transportation cost; ***p***_***i***_ denotes the purchase cost.

$$\begin{array}{l} |x-z_{i}|\in \{\tilde{x},1-\tilde{x}\}.\text{ To simplify the model},\text{let }k=1 \end{array}$$

If homebuyers get the same utility when they buy ordinary buildings or green housings, let

$$\begin{array}{l} U_{1}=U_{2}\\ V+w_{1}-t\tilde{x}-p_{1}=V+w_{2}-\;t\left(1-\tilde{x}\right)-p_{2} \end{array}$$

It can be obtained:

$$\begin{array}{l} \tilde{x}=\frac{t+\left(p_{2}-p_{1}\right)+\left(w_{2}-w_{1}\right)}{2t}=\frac{1}{2}-\frac{w_{2}-w_{1}}{2t}-\frac{p_{1}-p_{2}}{2t} \end{array}$$

That is to say, the homebuyers who are on the left side of $\tilde{x}$ choose the green housing, while the homebuyers on the right side of $\tilde{x}$ choose ordinary building.

Correspondingly, the demand functions are:

$$\begin{array}{r} D_{1}\left(p_{1},p_{2},w_{1},w_{2}\right)=N\tilde{x}=\left(\frac{1}{2}-\frac{w_{2}-w_{1}}{2t}-\frac{p_{1}-p_{2}}{2t}\right)N\\ D_{2}\left(p_{1},p_{2},w_{1},w_{2}\right)=N\left(1-\tilde{x}\right)=\left(\frac{1}{2}-\frac{w_{1}-w_{2}}{2t}-\frac{p_{2}-p_{1}}{2t}\right)N \end{array}$$

Then the profit of the green housing market:

$$\begin{array}{l} W_{1}=D_{1}\left(p_{1}-c_{1}\right) \end{array}$$

Profit of general construction market:

$$\begin{array}{l} W_{2}=D_{2}\left(p_{2}-c_{2}\right) \end{array}$$

Let$\frac{\partial W_{1}}{\partial p_{1}}=0\text{,}\frac{\partial W_{2}}{\partial p_{2}}=0\text{,}$ the Nash equilibrium prices are:

$$\begin{array}{l} p_{1}^{*}=t+\frac{{\text{w}}_{1}-w_{2}}{3}+\frac{2c_{1}+c_{2}}{3}\\ p_{2}^{*}=t+\frac{{\text{w}}_{2}-w_{1}}{3}+\frac{c_{1}+2c_{2}}{3} \end{array}$$

In the equilibrium state, the number of people buying green housings and ordinary buildings are respectively:

$$\begin{array}{l} {\text{D}}_{1}^{*}=(\frac{1}{2}-\frac{{\text{w}}_{2}-{\text{w}}_{1}}{6\text{t}}-\frac{{\text{c}}_{1}-{\text{c}}_{2}}{6\text{t}})\text{N}\\ {\text{D}}_{2}^{*}=(\frac{1}{2}+\frac{{\text{w}}_{2}-{\text{w}}_{1}}{6\text{t}}+\frac{{\text{c}}_{1}-{\text{c}}_{2}}{6\text{t}})\text{N} \end{array}$$

The equilibrium profits are

$$\begin{array}{l} ({\text{p}}_{1}^{*}-{\text{c}}_{1}){\text{D}}_{1}^{*}{(p}_{2}^{*}-{\text{c}}_{2})D_{2}^{*}. \end{array}$$

### The One Stage Model With Uncertainties

The basic model assumes that all house buyers have the same cost sensitivity in terms of transportation time, energy, and expenditure. However, in the actual situation, in the long run, some home buyers regret buying buildings far from the urban area, and some home buyers do not care, so they should be divided into different types. Homebuyers who are more sensitive to traffic costs use the type of high traffic costs to refer to, while those who are not sensitive to traffic costs use the type of low traffic costs to refer to. Therefore, in the one-stage model with uncertainties, it is assumed that:

(1) There are two types of homebuyers: High transportation cost type marked as $\bar{\text{t}}$, and probability is r; low transportation cost type marked as t, and probability is 1–r.
(2) Homebuyers are not clear about their types, but they have prior knowledge of r, which is represented by random variable ***r***^***B***^, whose value range is [0,1].

Assuming ***r***^***B***^ is the Beta distribution with parameters of (**α, β**), then the expectation of *r*^*B*^ is $E\left(r^{B}\;\right)=\frac{\alpha }{\alpha +\beta }$**.**

Let E(t) be the expectation of transportation cost obtained by buyers based on prior knowledge, so

$$\begin{array}{l} E\left(t\right)=E\left(r^{B}\right)\bar{t}+\left(1-E\left(r^{B}\right)\right)\underline{t}=\underline{t}+\frac{\alpha }{\alpha +\beta }\left(\bar{t}-\underline{t}\right) \end{array}$$

Corresponding demand functions are:

$$\begin{array}{r} D_{1}\left(p_{1},p_{2}\right)=N\tilde{x}=\left(\frac{1}{2}-\frac{w_{2}-w_{1}}{2E\left(t\right)}-\frac{p_{1}-p_{2}}{2E\left(t\right)}\right)N\\ D_{2}\left(p_{1},p_{2}\right)=N\left(1-\tilde{x}\right)=\left(\frac{1}{2}-\frac{w_{1}-w_{2}}{2E\left(t\right)}-\frac{p_{2}-p_{1}}{2E\left(t\right)}\right)N \end{array}$$

### The Two-Stage Model Based on Homebuyer Comment

This section considers the influence of the homebuyers' comment on the number of buyers and the stakeholders' decisions in the second stage.

After the first stage, buyers with high transportation costs who are not satisfied will choose another building in the second stage. If the number of homebuyers who change their choice in the second stage is defined in the interval [$x_{\underline{t}},x_{\bar{t}}$], and expectation of the transportation cost of homebuyers based on their prior knowledge is E(t), so according to the results of the above Hotelling model, the critical points can be obtained:

$$\begin{array}{r} \text{x}=\frac{1}{2}-\frac{{\text{w}}_{2}-{\text{w}}_{1}}{2\underline{t}}-\frac{{\text{p}}_{1}-{\text{p}}_{2}}{2\underline{t}}\\ {\text{x}}_{\bar{\text{t}}}=\frac{1}{2}-\frac{{\text{w}}_{2}-{\text{w}}_{1}}{2\bar{\text{t}}}-\frac{{\text{p}}_{1}-{\text{p}}_{2}}{2\bar{\text{t}}}\\ {\text{x}}_{\text{E}(\text{t})}=\frac{1}{2}-\frac{{\text{w}}_{2}-{\text{w}}_{1}}{2\text{E}(\text{t})}-\frac{{\text{p}}_{1}-{\text{p}}_{2}}{2\text{E}\left(\text{t}\right)} \end{array}$$

In the second stage, buyers update their understanding of their own type according to the bad comment in the first stage:

In the interval $[x_{\underline{t}},x_{\bar{t}}$], the expected number of buyers who change choices is:

$$\begin{array}{l} M=N\left(x_{\bar{t}}-x\right)=\frac{1}{2}\left(\frac{1}{\underline{t}}-\frac{1}{\bar{t}}\right)[\left({\text{w}}_{2}-w_{1}\right)+(p_{1}-p_{2})]N \end{array}$$

As can be seen from the above equation, the number of homebuyers who are subjected to adverse comment and change types is determined by the difference in psychological distance and price between the two kinds of buildings.

In the second stage, the prior probability is updated according to Bayesian method after the number changes:

$$\begin{array}{l} t^{B}=\{\begin{array}{c} \bar{\begin{array}{c} t \end{array}}\\ \underline{t} \end{array}\;\frac{The\;probability\;is\;updated\;as\;\frac{\alpha +M_{1}}{\alpha +\beta +M}}{The\;probability\;is\;updated\;as\;\frac{\beta +M-M_{1}}{\alpha +\beta +M}} \end{array}$$

The equilibrium of the two-stage model is solved by backward induction.

The equilibrium result of the second stage is:

(1) The profit of the green housing market is:

$$\begin{array}{l} W_{1}^{\prime \prime }=D_{1}^{\prime \prime }\left(p_{1}^{\prime \prime }-c_{1}\right)=N\left(\frac{1}{2}-\frac{w_{2}-w_{1}}{6{E\left(t\right)}^{\prime \prime }}-\frac{c_{1}-c_{2}}{6{E\left(t\right)}^{\prime \prime }}\right)\\ \;\left[{E\left(t\right)}^{\prime \prime }+\frac{w_{1}-w_{2}}{3}+\frac{c_{2}-c_{1}}{3}\right] \end{array}$$

(2) The profit of the ordinary construction market is:

$$\begin{array}{l} W_{2}^{\prime \prime }=D_{2}^{\prime \prime }\left(p_{2}^{\prime \prime }-c_{2}\right)=N\left(\frac{1}{2}+\frac{w_{2}-w_{1}}{6{E\left(t\right)}^{\prime \prime }}+\frac{c_{1}-c_{2}}{6{E\left(t\right)}^{\prime \prime }}\right)\\ \;\left[{E\left(t\right)}^{\prime \prime }+\frac{w_{2}-w_{1}}{3}+\frac{c_{1}-c_{2}}{3}\right] \end{array}$$

Thereinto ${E\left(t\right)}^{\prime \prime }=\underline{t}+\frac{\alpha +Mr}{\alpha +\beta +M}\left(\bar{t}-\underline{t}\right)$

Stage I equilibrium is as follows.

If the price of an ordinary building is the same as that of green housing, this means the price of an ordinary building is *p*_2_ = *E*(*t*). But if the price of a green housing rises (the decrease of subsidy also means the price rising) to *p*_1_ = *E*(*t*)+δ,(δ>0), Then the demand of the green housing market will change from $\left(\frac{1}{2}-\frac{w_{2}-w_{1}}{2\text{E}(t)}\right)N$ to $(\frac{1}{2}-\frac{w_{2}-w_{1}}{2\text{E}\left(t\right)}-\frac{\delta }{2E(t)})N$ (as shown above), and the demand will decrease by $\frac{\delta }{2E(t)}N$, here $M=\frac{1}{2}\left(\frac{1}{\underline{t}}-\frac{1}{\bar{t}}\right)\left(w_{2}-w_{1}+\delta \;\right)N.$

The profit difference between the two stages of green housing market is calculated as:

$$\begin{array}{l} {W_{1}}^{\prime \prime }-{W_{1}}^{\prime }=\left[\frac{{E\left(t\right)}^{\prime \prime }}{2}-\frac{{\left(w_{1}-w_{2}\right)}^{2}}{6{E\left(t\right)}^{\prime \prime }}-\frac{{E\left(t\right)}^{\prime }}{2}+\frac{{\left(w_{1}-w_{2}\right)}^{2}}{6{E\left(t\right)}^{\prime }}\right]N \end{array}$$

Thereinto

$$\begin{array}{r} {E\left(t\right)}^{\prime \prime }=\;\underline{t}+\frac{\alpha +Mr}{\alpha +\beta +M}\left(\bar{t}-\underline{t}\right)\\ {E\left(t\right)}^{\prime }=\;\underline{t}+\frac{\alpha }{\alpha +\beta }\left(\bar{t}-\underline{t}\right) \end{array}$$

Then,

$$\begin{array}{l} {W_{1}}^{\prime \prime }-W_{1}^{\prime }=[\frac{1}{2}\left(\bar{t}-\underline{t}\right)\left(\frac{\alpha +Mr}{\alpha +\beta +M}-\frac{\alpha }{\alpha +\beta }\right)+\frac{{\left(w_{1}-w_{2}\right)}^{2}}{6}\\ \;\left(\frac{1}{\underline{t}+\frac{\alpha }{\alpha +\beta }\left(\bar{t}-\underline{t}\right)}-\frac{1}{\underline{t}+\frac{\alpha +Mr}{\alpha +\beta +\text{M}}\left(\bar{t}-\underline{t}\right)}\right)]\\ \;\left[\begin{array}{c} \frac{1}{2}\left(\bar{t}-\underline{t}\right)\left(\frac{\alpha +r\cdot \frac{N}{2}\left(\frac{1}{\underline{t}}-\frac{1}{\bar{t}}\right)\left(w_{1}-w_{2}+\delta \right)}{\alpha +\beta +\frac{N}{2}\left(\frac{1}{\underline{t}}-\frac{1}{\bar{t}}\right)\left(w_{1}-w_{2}+\delta \right)}-\frac{\alpha }{\alpha +\beta }\right)+\frac{{\left({\text{w}}_{1}-w_{2}\right)}^{2}}{6}\cdot \\ \frac{\left(\bar{t}-\underline{t}\right)\left(\frac{\alpha +r\cdot \frac{N}{2}\left(\frac{1}{\underline{t}}-\frac{1}{\bar{\text{t}}}\right)\left(w_{1}-w_{2}+\delta \right)}{\alpha +\beta +\frac{N}{2}\left(\frac{1}{\underline{t}}-\frac{1}{\bar{\text{t}}}\right)\left(w_{1}-w_{2}+\delta \right)}-\frac{\alpha }{\alpha +\beta }\right)}{\left(\underline{t}+\frac{\alpha }{\alpha +\beta }\left(\bar{\text{t}}-\underline{t}\right)\right)\left(\underline{t}+\frac{\alpha +r\cdot \frac{N}{2}\left(\frac{1}{\underline{t}}-\frac{1}{\bar{\text{t}}}\right)\left(w_{1}-w_{2}+\delta \right)}{\alpha +\beta +\frac{N}{2}\left(\frac{1}{\underline{t}}-\frac{1}{\bar{\text{t}}}\right)\left(w_{1}-w_{2}+\delta \right)}\left(\bar{t}-\underline{t}\right)\right)} \end{array}\right]\text{N}\\ \;=N\left(\bar{t}-\underline{t}\right)\left(\frac{\alpha +Mr}{\alpha +\beta +\text{M}}-\frac{\alpha }{\alpha +\beta }\right)[\frac{1}{2}+\frac{{\left(w_{1}-{\text{w}}_{2}\right)}^{2}}{6}\cdot \\ \;\frac{1}{(\underline{t}+\frac{\alpha }{\alpha +\beta }\left(\bar{t}-\right)\underline{t})(\underline{t}+\frac{\alpha +\text{M}\text{r}}{\alpha +\beta +\text{M}}\left(\bar{t}-\underline{t}\right))}] \end{array}$$

Because $N\left(\bar{t}-\underline{t}\right)\left[\frac{1}{2}+\frac{{\left({\text{w}}_{1}-w_{2}\right)}^{2}}{6}\cdot \frac{1}{\left(\underline{t}+\frac{\alpha }{\alpha +\beta }\left(\bar{t}-\underline{t}\right)\right)\left(\underline{t}+\frac{\alpha +Mr}{\alpha +\beta +\text{M}}\left(\bar{t}-\underline{t}\right)\right)}\right]>0$, the positive and negative of this equation depends on $\frac{\alpha +Mr}{\alpha +\beta +M}-\frac{\alpha }{\alpha +\;\beta }$.

If $\frac{\alpha +\text{r}\cdot \frac{\text{N}}{2}\left(\frac{1}{\underline{t}}-\frac{1}{\bar{\text{t}}}\right)\left({\text{w}}_{2}-{\text{w}}_{1}+\delta \right)}{\alpha +\beta +\frac{\text{N}}{2}\left(\frac{1}{\underline{t}}-\frac{1}{\bar{\text{t}}}\right)\left({\text{w}}_{2}-{\text{w}}_{1}+\delta \right)}-\frac{\alpha }{\alpha +\beta }$ is positive, in the second stage, the profit of the green housing market increases compared with that of the first stage. If it is negative, the profit decreases. According to limit theory, when *w*_2_ − *w*_1_ + δ is small and $r>\frac{\alpha }{\alpha +\beta }$, the profit increases, while if $r<\frac{\alpha }{\alpha +\beta }$ the profit decreases. Because δ > 0, if *w*_2_ − *w*_1_ tends to −δ, then the green housing market has an incentive to change the expected price. That is to say, among the buyers with a high proportion of price sensitive ones, the difference between buyers' psychological distance for green housings and their psychological distance for ordinary buildings is the same as the difference between the price of green housings and ordinary buildings. The profit of the green housing market can still increase after the price rises. This shows that the psychological distance of home buyers occupies a large proportion of motivation in the green housing consumption market. Even if the price of green housing rises, as long as the psychological distance difference of green housing is higher than that of ordinary buildings, home buyers will still choose green housing.

## Conclusions

### Conclusions of the Study

First, the survey data were used to verify the influence factors of green housing choice behavior, and on this basis the buyers' two-stage choice model was built. The results indicate that buyers' psychological distance plays a bigger role in the green housing market, so in the promotion of green housing, the government or the construction enterprise should increase the publicity, so that the buyers will shorten their psychological distance to green housings.

The comments of home buyers can affect the purchase intention of green housing. Combined with the actual situation, specific recommendations are proposed as follows:

(1) Management of comment quality

The quality of online comments has a significant impact on green housing purchase intention, and high-quality comments help consumers to quickly identify information and generate high credibility for products, thus improving their willingness to buy. Therefore, merchants should deal with low-quality information in time, and make efforts in feedback communication with buyers, such as providing gifts or free after-sales service to prevent malicious comments. Establish a user reporting system, merchants and buyers together supervise the network water army and report quickly to prevent them from sending malicious comments.

(2) Management of comment titer

When the comment titer is positive, the purchase intention of green housing increases, and when the comment titer is negative, the purchase intention of green housing may decrease. Merchants cannot retain positive comments for product sales and massively delete negative comments. We should strengthen the diffusion of positive comments, express gratitude to buyers who publish positive comments in time through gifts and so on. Meanwhile, respond to buyers who publish negative comments quickly, as problem analysis and solutions are more necessary.

(3) Management of evaluator credibility

Establish a complete user comment system to ensure the reliability of information sources. Raise the registration threshold of evaluators, prevent a malicious water army; establish a relevant legal system, if malicious comments damage the reputation of merchants and result in economic losses, the legal responsibility can be investigated; set the rating of evaluators, users can judge the reliability of the comments according to the rating of evaluators, merchants can also give priority to the recommendations of senior evaluators to achieve the effect of product sales increasing.

(4) Suggestion from homebuyer perspective

Before visiting the comments, buyers should improve their awareness of green housing, so as to be able to screen useful information more quickly and efficiently and improve their ability to judge and reduce blind consumption; reasonably use comments to make correct purchase decisions and improve satisfaction.

(5) Suggestion from a government perspective

Enhance the popularization of green housing knowledge and improve the national cognition. Consumer's psychological distance is an important factor in green housing choice behavior. The citizens have too little an understanding of green housing, and cannot clearly understand the concept and advantages of green housing, which will affect the development of the green housing industry. The government and construction enterprise need to take measures to strengthen the publicity of green housing, improve its popularity, and make the citizens clearly understand the environmental protection of green housing and the necessity of its development, such as advertising and representative green housing development.

### Limitations of the Study

This article categorizes the evaluation of home buyers based on previous scholars' research, and divides them into four dimensions: comment quantity, comment quality, comment titer, and evaluator credibility as independent variables, but there are also studies showing that the dimensions of online evaluation are more than these. Therefore, in the future, we will continue to select other variables to study the influence mechanism of home buyers' evaluation on green housing purchase intention.

## Data Availability Statement

The raw data supporting the conclusions of this article will be made available by the authors, without undue reservation.

## Ethics Statement

The studies involving human participants were reviewed and approved by the Ethics Review Board of Business School in University of Jinan, China. Participants provided their written informed consent to participate in the study.

## Author Contributions

QF: conceptualization, resources, methodology, writing—original draft preparation, and supervision. XS: project administration, formal analysis, funding acquisition, and supervision. CC: data curation, writing—reviewing and editing. ZD: software, visualization, and investigation. YW: combing the literature, data recalculation, and writing—reviewing and editing. All authors contributed to the article and approved the submitted version.

## Conflict of Interest

The authors declare that the research was conducted in the absence of any commercial or financial relationships that could be construed as a potential conflict of interest.
